# Prelicensure Nursing Students’ COVID-19 Attitude Impact on Nursing Career Decision during Pandemic Threat in Taiwan: A Cross-Sectional Study

**DOI:** 10.3390/ijerph18063272

**Published:** 2021-03-22

**Authors:** Shu-Chun Lin, Lee-Fen Ni, Yu-Ming Wang, Shu Hsin Lee, Hung-Chang Liao, Cheng-Yi Huang, Ying-Chen Tseng

**Affiliations:** 1 Department of Nursing, Chang Gung University of Science and Technology, Taoyuan City 33303, Taiwan; sclin@mail.cgust.edu.tw (S.-C.L.); lfni@mail.cgust.edu.tw (L.-F.N.); 2 Chang Gung Memorial Hospital, Taoyuan City 33303, Taiwan; 3 Department of Psychology, Chung Shan Medical University, Taichung City 40201, Taiwan; wym@csmu.edu.tw; 4 Clinical Psychological Room, Chung Shan Medical University Hospital, Taichung City 40201, Taiwan; 5 School of Nursing, Chung Shan Medical University, Chung Shan Medical University Hospital, Taichung City 40201, Taiwan; shl@csmu.edu.tw (S.H.L.); ycyct73@gmail.com (Y.-C.T.); 6 Department of Nursing, Chung Shan Medical University Hospital, Taichung City 40201, Taiwan; 7 Department of Health Services Administration, Chung Shan Medical University, Taichung City 40201, Taiwan; hcliao@csmu.edu.tw; 8 Department of Medical Education, Chung Shan Medical University Hospital, Taichung City 40201, Taiwan

**Keywords:** nursing shortage, career attitudes, career decision-making, attitude scale, pandemic threat, prelicensure nursing students

## Abstract

The COVID-19 pandemic may cause a nursing shortage. Prelicensure nursing students who are exposed to high-stress COVID-19 events are related to defective career decision-making. This study validated the COVID-19 attitude scale and clarified how their attitudes about COVID-19 affected their behavioral intentions toward career decision-making. We conducted a cross-sectional study and recruited a convenience sample of 362 prelicensure nursing students from Northern and Central Taiwan. Two measurements were applied, including the Nursing Students Career Decision-making instrument and COVID-19 attitude scale. We used AMOS (version 22.0) to perform a confirmatory factor analysis. The Cronbach α of the COVID-19 attitude scale was 0.74 and consisted of four factors. The most positive attitude was the nursing belief factor, and the least positive factor was emotional burden. Prelicensure nursing students’ COVID-19 attitudes were significantly positively associated with their career decision-making attitudes and perceived control (ß = 0.41 and ß = 0.40, respectively; *p* < 0.001). All the key latent variables explained significantly 23% of the variance in the career decision-making behavioral intentions module. In conclusion, the COVID-19 attitude scale is valid. Although the prelicensure nursing students’ COVID-19 attitudes had no direct effect on career decision-making intentions, they had a direct effect on career decision-making attitudes and the perceived control.

## 1. Introduction

COVID-19 is a novel infectious disease that carries unprecedented, substantial morbidity and mortality rates in the human population [1,2]. At the time of publication, more than 50 million confirmed cases of COVID-19, including over one million deaths, have been reported to the World Health Organization, and the cases increase continually [3]. Providing care to infected patients carries an extensive contact risk because of continued exposure to critically ill patients with apparently high viral shedding levels [4]. Nurses suffer from unprecedented psychological stress and anxiety and endured trauma, causing mental disorders [5,6,7]. The COVID-19 pandemic has placed extraordinary pressure and threat on the nursing workforce, thereby abruptly and profoundly affecting clinical nursing and nursing education [8], and the perceived threat of COVID-19 is significant in explaining burnout in nurses [9]. As the risks and effects of COVID-19 on nurses become increasingly apparent, nurses leave the workplace early due to physical and psychological stress, which exacerbates the nurses’ shortage [10]. The workplace burnout conditions may have an impact on career decision-making for prelicensure nursing students. Senior nursing students have clinical practice courses; although a preceptor supervises them, many students fear contracting the virus [11]. Prelicensure nursing students exposed to high-stress COVID-19 events are continually retraumatized; these bad experiences are related to defective decision-making and harmful ways of behaving in adulthood [12].

In the global COVID-19 pandemic, caring providers were, and continue to be, on the “front lines”, taking care of patients and placing themselves and their families in danger of contracting the virus (SARS-CoV-2) [13]. Nurses have been traumatized and emotionally affected by the Covid-19 pandemic, and prelicensure nursing students are not invulnerable to these traumatic experiences. In their formative years of nursing education, some practicing nursing students have been in the position of deciding between the wellbeing of their family and the best track for the near future of their careers [12]. The primary emotions and perceptions of prelicensure nursing students in the pandemic include anxiety, fear of possible infection and transmission, uneasiness and severe concern [8,14]. Conversely, for some nursing students, entering the nursing profession has been reinforced during the COVID 19 pandemic [15], or they disclosed that they would take on the risk of acquiring COVID-19 because of the health of patients and communities [16]. The nursing and medical students described their reasons for volunteering to work during the pandemic were the need for help given the current situation, moral obligation, altruistic and vocational aspects of working, humanitarianism and a sense of cooperation [8]. Despite their fear of becoming infected with the virus, many prelicensure nursing students wished to help address the health catastrophe. However, some such nursing or medical students did not join the healthcare system due to the fear of infection and transmission to their family, not having sufficient personal protection equipment, and being unprepared for the current emergency working situation [11]. We have no way of knowing prelicensure nursing students’ attitudes concerning COVID-19, because there is no relevant scale for us to explore. The unprecedented global health impact of the pandemic launched a new era for career decision-making for nursing students.

The theory of planned behavior developed by Ajzen proposes that the determinants of behavior comprise the intent to carry out specific behaviors and that intention is influenced by attitude and subjective or perceived behavioral control [17]. According to this theory, more positive attitudes, reduced stress from subjective norms and heightened behavioral control awareness enhance behavioral intentions [17]. The theory of planned behavior has served as the theoretical framework in career choices, and there is strong evidence of the aspects of development and attitude, subjective norms and perceived behavioral control supporting occupational intentions [18,19]. A study following the 2003 severe acute respiratory syndrome (SARS) outbreak in Taiwan demonstrated that attitude, subjective norms and self-efficacy could effectively predict nursing students’ behavioral intentions of caring for SARS patients [20]. Lin (2010) indicated the Career Decision- Making (CDM) attitude toward the behavior, the subjective norm of the behavior and the perceived behavior control could affect the CDM behavior intentions of nursing undergraduate students in Taiwan [21]. Lin and Liu further found that department or school factors could moderate the CDM perceived behavioral control affecting the CDM behavior intentions of nursing undergraduate students. This study aimed to develop the COVID-19 attitude scale and understand the attitude, subjective norms and perceived control of career decision-making among prelicensure nursing students during the period when COVID-19 was active in Taiwan. We also sought to clarify how their attitudes about COVID-19 affected their behavioral intentions.

### Research Model and Hypotheses Development

**Figure 1:**
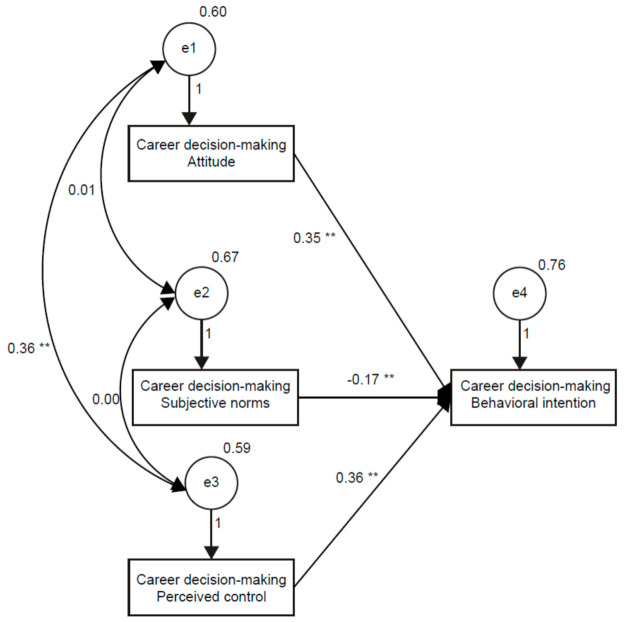
Structural path model of career decision-making attitude, subjective norms, perceived control and behavioral intention with standardized coefficients. Note: ** *p* < 0.01. CDM: career decision-making.

The prelicensure nursing students’ career decision-making behavioral intention model has been validated [22]. In this study, we examined the effects of students’ attitudes toward COVID-19 in this hypothetical model, shown in Figure 1. Based on this model, we proposed the following hypotheses:

**Hypothesis** **1:** *Career decision-making attitude will be positively associated with career decision-making behavioral intention.*

**Hypothesis** **2:** *The subjective norms of career decision-making will be negatively associated with career decision-making behavioral intention.*

**Hypothesis** **3:** *Perceived control of career decision-making will be positively associated with career decision-making behavioral intention.*

**Hypothesis** **4a:** *COVID-19 attitude will be moderate the relationship between attitude and career decision-making behavioral intention.*

**Hypothesis** **4b:** *COVID-19 attitude will be moderate the relationship between subjective norms and career decision-making behavioral intention.*

**Hypothesis** **4c:** *COVID-19 attitude will be moderate the relationship between perceived control and career decision-making behavioral intention.*

**Hypothesis** **5:** *COVID-19 attitude will be positively associated with career decision-making behavioral intention.*

## 2. Materials and Methods

### 2.1. Study Design and Procedure

This cross-sectional, descriptive, self-report study was conducted in Northern and Central Taiwan from May to August 2020. For the structural equation modeling (SEM) analysis, we calculated the sample size by using Free Statistics Calculators 4.0 (https://www.danielsoper.com/statcalc/calculator.aspx?id=89, accessed on 1 March 2020). The five input parameters were as follows: effect size, 0.3; preferred statistical power level, 0.8; number of latent variables, 5; number of observed variables, 11 and significance level, 0.05. Using convenience cluster sampling, we recruited 362 prelicensure nursing students as participants from two universities, one in Northern Taiwan and one in Central Taiwan. The minimum number of participants required for the model structure was 352; thus, our sample size of 362 students (return rate, 97%) was abundant. The inclusion criterion was participants who would graduate in July 2020. We recruited fourth-year junior nursing students who met the inclusion criterion to participate in the study. The researchers explained the purpose and process of the study to each class. The researchers also provided written information and oral explanations to participants. All participants provided informed consent. The researchers provided the study questionnaires to each participant and collected the data after class. The data of students who did not complete their questionnaires were excluded from the analysis.

### 2.2. Measurements

Two measurements were applied, including the Nursing Students Career Decision-making Instrument (NSCDM) and COVID-19 attitude (COVID-A) scale.

#### 2.2.1. Nursing Students Career Decision-Making Instrument (NSCDM)

Nursing students’ career decision-making behavior was measured using 25 questions from the NSCDM [22]. This instrument has four dimensions: decision-making attitude (8 items), subjective norms (10 items), perceived control (10 items) and behavioral intention (5 items). Every participant can express how much they agree or disagree with each item using a 6-point Likert-type scale (1 = completely inconsistent to 6 = entirely consistent). Items 2, 3 and 4 of the behavioral intention dimension and item 6 of the attitude dimension are reverse questions; they are reverse-scored. A higher score indicates a more positive attitude, tremendous pressure of subjective norms and more substantial perceived behavior competence. The original study reported that Cronbach’s α of the subscales ranged from 0.75 to 0.91, and Cronbach’s α for the whole instrument was 0.92. Construct validity was analyzed by exploratory factor analysis (EFA), factor loadings higher than 0.40 and accounted variance ranged from 53.72% to 70.99% [22].

#### 2.2.2. COVID-19 Attitude (COVID-A) Scale

The nursing student’s COVID-19 attitudes were measured using the COVID-A scale. The authors developed the COVID-A scale from a broad reference study and semi-structured meaningful conversation with ten prelicensure nursing students. We constructed 20 items initially, and then, the scale was validated by content validity, Cronbach’s α and evaluation of construct validity. We invite three specialized experts in nursing education, psychology and hospital clinical management to evaluate the content validity. They evaluated each item on a four-point ordinal scale in term of its simplicity, relevance and suitability. All items were designed on a seven-point Likert scale based on the individual degree of agreement with the items: 1 = strongly disagree, 2 = really disagree, 3 = somewhat disagree, 4 = neutral, 5 = somewhat agree, 6 = really agree and 7 = strongly agree. The overall Cronbach’s α of the 20- item instrument was 0.74, and the Cronbach’s α of the four dimensions ranged from 0.82 to 0.90, indicating good internal consistency and reliability (Table 1).

Construct validity was examined by the EFA, which is conducted to explore the phenomenon underlying element [23]. We extracted the main factor using principal component analysis and varimax rotation.

We preserved an item if its factor loading was larger than 0.60 on the relevant factors and smaller than 0.40 on the nonrelevant factors [24]. The screen plot of the eigenvalues (≥1) and the Kaiser–Meyer–Olkin (KMO) test were applied to determine the number of factors of the scale. The KMO value was 0.849, which was higher than the threshold value of 0.6 [25]. Bartlett’s test of sphericity was significant (chi-square = 4173.917; *p* = 0.000 < 0.05). The KMO and Bartlett’s values confirmed the suitability of the sample size for the EFA. The four factors accounted for 67.01% of the variance. This instrument has four dimensions: emotional burden (Factor 1: 6 items), nursing belief (Factor 2: 5 items), institution protective support (Factor 3: 5 items) and hazard of COVID-19 (Factor 4: 4 items).

### 2.3. Data Analyses

We analyze data by using IBM SPSS Statistics for Windows, version 22.0. (IBM Corp., Armonk, NY, USA). The gender proportion is presented as counts and percentages, while age is presented as means and standard deviation (mean ± SD). The results of a series of independent *t*-tests were applied to examine the information on career decision-making attitude, subjective norms, perceived control and behavioral intention and COVID-19 attitude. The study adopted the EFA to inspect the structure of the COVID-19 attitude scale. Then, we used AMOS (version 22.0, IBM Corp., Armonk, NY, USA) to perform a confirmatory factor analysis to check whether participants’ information from the construct measurements was in line with our hypothetical model. Many fit indices were employed to determine the research architecture model, including the likelihood ratio, X^2^/df ratio (CMIN/DF), the goodness of fit (GFI), adjusted goodness of fit (AGFI), standardized root mean square residual (SRMR), root mean square error of approximation (RMSEA), normed fit index (NFI), non-normed fit index (NNFI), relative fit index (RFI), incremental fit index (IFI) and comparative fit index (CFI).

### 2.4. Ethics and Consent

The Institutional Review Board of Chung Shan Medical University Hospital ethics committee approved this study (No: CS1-20055). We obtained administrative assistance from the two universities’ nursing schools. First, we applied to the school’s research and development management unit to initiate the project. After obtaining permission to proceed with the study, we contacted class representatives and arranged meetings with students after class. At this stage, we explained the purpose and process of the study to the students. Those who agreed to participate received the questionnaire only after providing written informed consent. The eligibility criterion was prelicensure nursing students, and we excluded those who had not practiced in the hospital during the semester before the data were collected.

## 3. Findings

### 3.1. Participants’ Characteristics

The participants’ ages ranged from 22 to 24 years, and the average ages for School A and School B were 21.46 ± 0.83 and 21.40 ± 1.79, respectively (P/X^2^ = 0.70), with no significant differences between the two schools. The proportion of women in the final sample was 87.5%.

### 3.2. Relationship among Latent Variables and Model Testing

The mean of the total scores of the COVID-19 attitudes ranged from 59–133 (89.3 ± 10.49), with higher scores indicating a positive attitude toward the COVID-19 threat. The most positive factor was the nursing belief factor (mean of item scores = 5.0 ± 0.93); then, the means gradually decreased. Sequentially, they were as follows: hazard of COVID-19 (4.91 ± 1.16), institution protective support (4.89 ± 0.86) and emotional burden (3.38 ± 1.18). Five latent variables—career decision-making attitude, subjective norms, perceived control, behavioral intention and COVID-19 attitude—were included in the measurement model. Table 2 shows the convergent validity (average variance extracted, AVE), composite reliability (CR), square root of AVE (√AVE) and correlation coefficients (r) between variables. The AVE of each latent variable was larger than 0.36, which is an acceptable value [26]. The CR of each latent variable was over 0.70, which is an adequate value [27]. The Cronbach’s α for all factors were above 0.70, indicating that the measures had good reliability [28]. Discriminant validity was confirmed if the calculated values of the square root of the AVE of factors were higher than the correlation coefficient (r) between the factors [26]. Table 2 also shows that the criterion is met with respect to the √AVE being greater than the highest correlation coefficient (r) between the factors. Furthermore, career decision-making attitude was positively correlated with career decision-making behavioral intention (r = 0.416, *p* < 0.01) (hypothesis 1), career decision-making perceived control (r = 0.628, *p* < 0.01) and COVID-19 attitude (r = 0.414, *p* < 0.01) but was not correlated with career decision-making subjective norms. Career decision-making subjective norms was negatively correlated with career decision-making behavioral intention (r = −0.130, *p* < 0.01)) (hypothesis 2) but was not correlated with career decision-making perceived control and COVID-19 attitude. Career decision-making perceived control was positively correlated with career decision-making behavioral intention (r = 0.412, *p* < 0.01) (hypothesis 3) and COVID-19 attitude (r = 0.404, *p* < 0.01). COVID-19 attitude was positively correlated with career decision-making behavioral intention (r = 0.223, *p* < 0.01) (hypothesis 5).

The confirmatory factor analysis was applied to examine the model (Table 3). The fit of the “non-COVID-19 attitude moderating model” was optimal without modification (likelihood ratio χ2 = 0.088, CMIN/DF (χ2/df) = 0.044, GFI = 1.000, AGFI = 0.999, SRMR = 0.010, RMSEA = 0.010, NFI = 1.000, NNFI = 1.000, RFI = 0.999, IFI = 1.000 and CFI = 1.000), which represents a good fit between the hypothetic model and the collected data.

### 3.3. Fitting the Proposed Model

**Figure 2:**
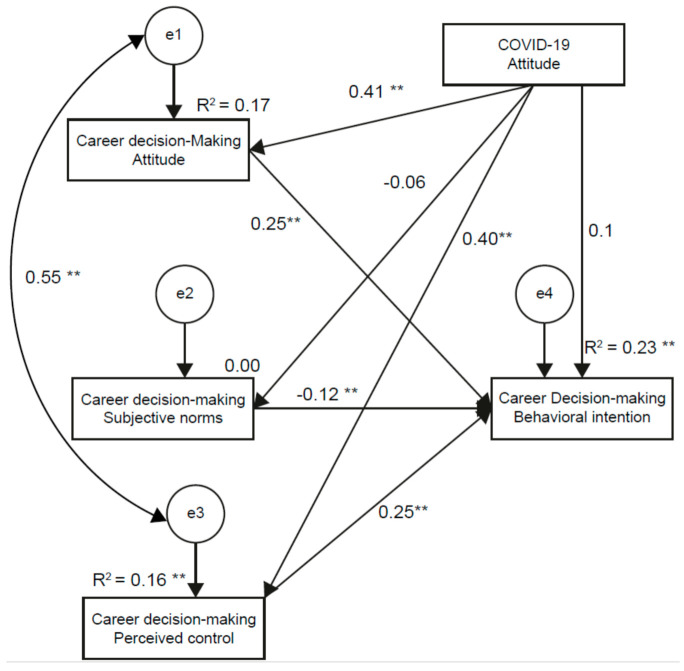
Structural path model of the COVID-19 attitude, career decision-making attitude, subjective norms, perceived control and behavioral intentions with standardized coefficients. Note: ** *p* < 0.01.

A full model was subsequently tested. Table 3 shows that the fit of the COVID-19 attitude moderating model was also optimal without modification (likelihood ratio χ2 = 1.419, CMIN/DF (χ2/df) = 0.069, GFI = 1.000, AGFI = 0.999, SRMR = 0.005, RMSEA = 0.001, NFI = 1.000, NNFI = 1.002, RFI = 0.999, IFI = 1.000 and CFI = 1.000). The display model fit was still good. Figure 1 presents the full structural path model with standardized coefficients, which postulated the direct paths between the latent variables. Five latent variables—career decision-making attitude, career decision-making subjective norms, career decision-making perceived control, career decision-making behavioral intention and COVID-19 attitude—were included in the measurement model. Figure 2 shows that career decision-making attitude and career decision-making perceived control were positively correlated with career decision-making behavioral intention (hypotheses 1 and 3) (ß = 0.25 and ß = 0.25, respectively; *p* < 0.001), while career decision-making subjective norm was significantly and negatively associated with career decision-making behavioral intention (hypothesis 2) (ß = −0.12; *p* < 0.001). COVID-19 attitude was not significantly correlated with career decision-making behavioral intention (hypothesis 5) (ß = −0.01; *p* > 0.05). COVID-19 attitude was significantly positively associated with career decision-making attitude and career decision-making perceived control (ß = 0.41 and ß = 0.40, respectively; *p* < 0.001) (hypotheses 4a and 4b) but was not significantly associated with career decision-making subjective norms (ß = −0.06; *p* > 0.05) (hypothesis 4c). Therefore, hypotheses 1, 2, 3, 4a and 4c were supported; Hypotheses 4b and 5 were not proven. These results point to a direct influence of COVID-19 attitude on career decision-making attitude and career decision-making perceived control. COVID-19 attitude indirectly affected career decision-making behavioral intention via career decision-making attitude and career decision-making perceived control. Ultimately, all the key latent variables and COVID-19 attitude explained significantly 23% of the variance in career decision-making behavioral intentions.

## 4. Discussion

### 4.1. The Coefficients of the Relationships between Latent Variables

The health damage and impacts of COVID-19 worldwide are still ongoing. The daily increase in the number of patients has increased the dilemma and pressure on nurses. Nursing students’ career choices consider the realization of their expectations and bear the risk of personal or family members being infected. In this cross-sectional study, we first examined the reliability and validity of measuring career decision-making and COVID-19 attitudes, which were all acceptable. Second, we examine the coefficient of the relationship between the latent variables. In line with past research on the theory of planned behavior and career issues, the core theory of planned behavior variables’ attitude, subjective norms and perceived behavioral control were all significantly (*p* < 0.01) correlated with the behavioral intention for the respondents [18,29]. The results showed that career decision-making attitudes and perceived control were significantly positively correlated with career decision-making behavioral intentions. The lower stress of the subjective norms significantly enhanced the formulation with the career decision-making behavioral intentions. These outcomes are consistent with the theory of planned behavior [17]. 

Furthermore, COVID-19 attitude was significantly positively associated with career decision-making attitude and behavioral intention but not significantly correlated with career decision-making subjective norms and intention. Hypotheses 4a and 4c were supported but Hypotheses 4b and 5 were not. This finding is not consistent with the behavioral intention of caring for SARS patients among nursing students [20]. These inconsistent results with previous studies reveal an important message: although students’ COVID-19 attitude does not directly affect their career decision-making intentions, the COVID-19 attitude is significantly related to the career decision-making attitude and perceived control. This finding seems to reveal that students are still worried about the threat of COVID-19, and even if they are afraid, they still have confidence in their nursing abilities and nursing work. Compared with the past, the poor control of SARS and defective equipment have caused nurses’ infections and sacrifices, causing nurses to refuse to participate in patients’ care. This time, the government and agencies have adopted advanced arrangements to prevent the spread of COVID-19. Therefore, although prelicensure nursing students are still a little emotionally anxious, their emotions will not affect their rational career decision-making. This finding may also speak positively to the Taiwanese government’s correct and effective performance in dealing with the COVID-19 pandemic. The government immediately adopted anti-epidemic measures to identify infected persons and contacts for their isolation or quarantine and provided medical staff with sufficient personal protective equipment (PPE), which has controlled the spread of the outbreak domestically [30]. These outstanding performances increased the confidence of prelicensure nursing students in entering the workplace.

### 4.2. Impacts of COVID-19 Attitude and Career Decision-Making Behavioral Intentions

Finally, we tested the effect of the COVID-19 attitude on senior nursing students’ career decision-making. In Figure 2, the SEM analysis revealed nursing students’ COVID-19 attitudes and career decision-making behavioral intentions. The final model shows that a positive career decision-making attitude, stronger perceived control and less subjective norms directly predict career decision-making behavioral intentions. These results are in line with previous studies indicating that the attitude, subjective norm and perceived behavioral control core variables were associated with career intentions significantly [18,19,29]. While the COVID-19 attitude had only an indirect effect, which is not consistent with the literature, we argue the COVID-19 pandemic may have placed extraordinary pressure on nurses; this pressure may lead to defective decision-making [12]. This result may be because the researched country has an outstanding performance in controlling the spread of outbreaks. The direct influence was flattened by the proactive preparedness and deployment by the government. Given the direct and indirect effects together, the model indicates that prelicensure nursing students’ career decision-making behavioral intentions can be enhanced by emphasizing the career decision-making attitude, comfort (rather than stress) from subjective norms from significant others and stronger infection control competence. Lin (2010) indicated the CDM attitude toward the behavior, the subjective norm of the behavior and the perceived behavior control could affect the CDM behavior intentions of nursing undergraduate students in Taiwan. We added that the COVID-19 attitude was not significantly correlated with the CDM behavioral intentions. Even though the epidemic is still prevalent in various countries, the lives of Taiwanese people have long returned to normal. Taiwan takes the lead with border control and uses technology to mobilize the entire Taiwanese epidemic prevention. So, although we found the nursing students’ COVID-19 attitudes directly affected their CDM attitudes and perceived control, their COVID-19 attitudes were not significantly correlated with their CDM behavioral intentions.

There are some limitations to this study. First, this study was conducted in Taiwan, where the spread of COVID-19 has effectively been controlled (at the time of this writing); hence, the burden of medical care was far lower than in other countries, so the impact of the COVID-19 threat cannot be generalized to other countries. Second, COVID-19 attitudes may be due to differences in culture or pandemic levels that would present a diversity impact. We recommend that further research on our model be conducted longitudinally and across different cultures.

## 5. Conclusions

Prelicensure nursing students’ COVID-19 attitudes have a direct effect on career decision-making attitude and perceived control. The COVID-19 pandemic has not yet ended, and nurses play a crucial role in combating this global crisis. The health delivery system requires a large number of registered nurses to join in front-line clinical care. From a nursing educational perspective, more courses about COVID-19 virus infection control knowledge and PPE application skills could be provided online, in nursing schools and through institutional continued education; online support groups can also be promoted at the institutional level. Nursing schools should implement education about protective equipment, infection control and mental health interventions for prelicensure nursing students since the outbreak’s initial phase. We believe that through clear and transparent information and sufficient resources, prelicensure nursing students can enhance their attitudes and confidence in choosing clinical care, reduce their subjective normative pressure from significant others and have a firm belief in entering clinical care. The healthcare system must have a sufficient professional workforce to fight this pandemic.

## Figures and Tables

**Table 1:** Rotated factor loading of the COVID-19 attitude scale.

Item	Factor of COVID-19 Attitude Scale			
(Attitude Code)	Factor 1 Emotional Burden	Factor 2 Nursing Belief	Factor 3 Institutional Protective Support	Factor 4 Hazard of COVID-19
	Factor 1: Cronbach’s α = 0.90			
CV-A 19	0.854			
CV-A 20	0.853			
CV-A 19	0.836			
CV-A 16	0.799			
CV-A 8	0.719			
CV-A 7	0.694			
	Factor 2: Cronbach’s α = 0.86			
CV-A 14		0.881		
CV-A 15		0.876		
CV-A 13		0.748		
CV-A 12		0.735		
CV-A 18		0.599		
	Factor 3: Cronbach’s α = 0.82			
CV-A 9			0.804	
CV-A 10			0.745	
CV-A 11			0.741	
CV-A 4			0.737	
CV-A 2			0.609	
	Factor 4: Cronbach’s α = 0.86			
CV-A 5				0.830
CV-A3				0.816
CVA6				0.797
CVA1				0.742
Eigenvalue	5.417	4.870	1.650	1.465
% of variance	20.80	17.44	14.53	14.25

Notes. Overall α = 0.74, total variance explained is 67.01 and CV-A: COVID-Attitude.

**Table 2:** Validity, reliability, square root of AVE (√AVE) and the matrix of correlations between variables in career decision-making for nursing students.

Variable	AVE	CR	α	Correlation				
				1	2	3	4	5
1. Career decision-making attitude	0.533	0.850	0.878	0.730				
2. Career decision-making subjective norms	0.482	0.821	0.758	−0.06	0.630			
3. Career decision-making perceived control	0.553	0.890	0.895	0.628 **	−0.08	0.730		
4. Career decision-making behavioral intention	0.473	0.771	0.721	0.416 **	−0.13 **	0.412 **	0.687	
5. COVID-19 attitude	0.600	0.950	0.740	0.414 **	−0.06	0.404 **	0.223 **	0.775

Note: α = Cronbach’s alpha. Diagonal components in the correlation column are the √ AVE values extracted for each latent variable. ** *p* < 0.01.

**Table 3:** Goodness-of-fit indices for the COVID-19 attitude moderating model.

Assessment Index for Model Fit	Criteria for Assessment	Non-COVID-19 Attitude Moderator Model		COVID-19 Attitude Moderator Model	
		Value of Index	Results of Assessment	Value of Index	Results of Assessment
Absolute model fit indices					
Likelihood Ratio χ2(*p*)	*p* > 0.05	0.088 (0.957)	Good	1.419 (0.934)	Good
CMIN/DF (χ2/df)	≤5	0.044	Good	0.069	Good
GFI	≥0.90	1.000	Good	1.000	Good
AGFI	≥0.90	0.999	Good	0.999	Good
SRMR	≤0.08	0.010	Good	0.005	Good
RMSEA	≤0.08	0.010	Good	0.001	Good
Incremental model fit indices					
NFI	≥0.90	1.000	Good	1.000	Good
NNFI	≥0.90	1.000	Good	1.002	Good
RFI	≥0.90	0.999	Good	0.999	Good
IFI	≥0.90	1.000	Good	1.000	Good
CFI	≥0.90	1.000	Good	1.000	Good

CMIN/DF = Minimum Discrepancy Function by Degrees of Freedom divided, df = degree of freedom, AGFI = adjusted goodness of fit, CFI = comparative fit index, GFI = goodness of fit, IFI= incremental fit index, NFI = normed fit index, NNFI = non-normed fit index, RFI = relative fit index, RMSEA = root mean square error of approximation and SRMR = standardized root mean square residual.

## Data Availability

The data that support the findings of this study are available on request from the corresponding author. The data are not publicly available due to privacy of research participants.

## References

[1] Perlman S. (2020). Another Decade, Another Coronavirus. N. Engl. J. Med..

[2] Pitman S. (2020). PTSD and Covid-19: A consequence of caring. World Ir. Nurs. Midwifery.

[3] World Health Organization (2021). WHO Coronavirus (COVID-19) Dashboard. https://covid19.who.int/.

[4] Arabi Y.M., Murthy S., Webb S. (2020). COVID-19: A novel coronavirus and a novel challenge for critical care. Intensiv. Care Med..

[5] Griffith R. (2020). Negligence, trauma and nervous shock. Br. J. Nurs..

[6] Hong S., Ai M., Xu X., Wang W., Chen J., Zhang Q., Wang L., Kuang L. (2020). Immediate psycho-logical impact on nurses working at 42 government-designated hospitals during COVID-19 outbreak in China: A cross-sectional study. Nurs. Outlook.

[7] Zheng R., Zhou Y., Fu Y., Xiang Q., Cheng F., Chen H., Xu H., Fu L., Wu X., Feng M. (2021). Prevalence and associated factors of depression and anxiety among nurses during the outbreak of COVID-19 in China: A cross-sectional study. Int. J. Nurs. Stud..

[8] Collado-Boira E.J., Ruiz-Palomino E., Salas-Media P., Folch-Ayora A., Muriach M., Baliño P. (2020). “The COVID-19 outbreak”—An empirical phenomenological study on perceptions and psychosocial considerations surrounding the immediate incorporation of final-year Spanish nursing and medical students into the health system. Nurse Educ. Today.

[9] Guixia L., Hui Z. (2020). A Study on Burnout of Nurses in the Period of COVID-19. Psychol. Behav. Sci..

[10] Spurlock D. (2020). The Nursing Shortage and the Future of Nursing Education Is in Our Hands. J. Nurs. Educ..

[11] Monforte-Royo C., Fuster P. (2020). Coronials: Nurses who graduated during the COVID-19 pandemic. Will they be better nurses?. Nurse Educ. Today.

[12] Fowler K., Wholeben M. (2020). COVID-19: Outcomes for trauma-impacted nurses and nursing students. Nurse Educ. Today.

[13] Swift A., Banks L., Baleswaran A., Cooke N., Little C., McGrath L., Meechan-Rogers R., Neve A., Rees H., Tomlinson A. (2020). COVID-19 and student nurses: A view from England. J. Clin. Nurs..

[14] Kochuvilayil T., Fernandez R.S., Moxham L.J., Lord H., Alomari A., Hunt L., Middleton R., Halcomb E.J. (2021). COVID-19: Knowledge, anxiety, academic concerns and preventative behaviours among Australian and Indian undergraduate nursing students: A cross-sectional study. J. Clin. Nurs..

[15] Santos L.M.D. (2020). The Relationship between the COVID-19 Pandemic and Nursing Students’ Sense of Belonging: The Experiences and Nursing Education Management of Pre-Service Nursing Professionals. Int. J. Environ. Res. Public Heal..

[16] Dewart G., Corcoran L., Thirsk L., Petrovic K. (2020). Nursing education in a pandemic: Academic challenges in response to COVID-19. Nurse Educ. Today.

[17] Ajzen I., Madden T.J. (1986). Prediction of goal-directed behavior: Attitudes, intentions, and perceived behavioral control. J. Exp. Soc. Psychol..

[18] Arnold J., Loan-Clarke J., Coombs C., Wilkinson A., Park J., Preston D. (2006). How well can the theory of planned behavior account for occupational intentions?. J. Vocat. Behav..

[19] Wilbourn M., Salamonson Y., Ramjan L., Chang S. (2017). Development and psychometric testing of the Attitudes, Subjective Norms, Perceived Behavioural Control, and Intention to Pursue a Career in Mental Health Nursing scale. Int. J. Ment. Health Nurs..

[20] Shen S.-F., Yeh G.-L. (2005). The Influencing Factors Toward Behavioral Intention of Caring SARS Patients among Bachelor Nursing Students. Educ. Stud..

[21] Lin S.C. (2010). The Study of Multilevel Model Construction on Vocational Decision Making Behavior Intention of Nursing Undergraduate Students in Taiwan. Ph.D. Thesis.

[22] Lin S.C., Liu H.F. (2012). The Development and Testing of Nursing Undergraduate Students’ Career Decision Making Behavior Intention Instrument. Chang Gung Care.

[23] Fabrigar L.R., Wegener D.T., Maccallum R.C., Strahan E.J. (1999). Evaluating the use of exploratory factor analysis in psychological research. Psychol. Methods.

[24] Stevens J. (1996). Applied Multivariate Statistics for the Social Sciences.

[25] Kaiser H.F. (1974). An index of factorial simplicity. Psychometrika.

[26] Fornell C., Larcker D.F. (1981). Evaluating Structural Equation Models with Unobservable Variables and Measurement Error. J. Mark. Res..

[27] Hair J.F., Hult G.T.M., Ringle C., Sarstedt M. (2016). A Primer on Partial Least Squares Structural Equation Modeling (PLS-SEM).

[28] Cronbach L.J. (1951). Coefficient alpha and the internal structure of tests. Psychometrika.

[29] Bai X.-L., Wang A.-N., Plummer V., Lam L., Cross W., Guan Z.-Y., Hu X., Sun M., Tang S.-Y. (2019). Using the theory of planned behaviour to predict nurse’s intention to undertake dual practice in China: A multicentre survey. J. Clin. Nurs..

[30] Chen S.-F., Huang L.-H., Chen C.-M., Chuang T.-H., Peng M.-T., Wang H.-H. (2020). The Key Role of Taiwanese Nurses in Combating COVID-19 Pandemic. Hu Li Za Zhi.

